# Parent and PHY Selection in Slot Bonding IEEE 802.15.4e TSCH Networks

**DOI:** 10.3390/s21155150

**Published:** 2021-07-29

**Authors:** Glenn Daneels, Dries Van Leemput, Carmen Delgado, Eli De Poorter, Steven Latré, Jeroen Famaey

**Affiliations:** 1 Internet Technology and Data Science Lab, University of Antwerp—imec, 2000 Antwerp, Belgium; glenn.daneels@uantwerpen.be (G.D.); steven.latre@uantwerpen.be (S.L.); 2 Internet Technology and Data Science Lab, Ghent University—imec, 9000 Ghent, Belgium; dries.vanleemput@ugent.be (D.V.L.); eli.depoorter@ugent.be (E.D.P.); 3 AI-Driven Systems, i2CAT Foundation, 08034 Barcelona, Spain; carmen.delgado@i2cat.net

**Keywords:** TSCH, slot bonding, multi-PHY, PHY selection, parent selection, routing, Contiki implementation

## Abstract

While IEEE 802.15.4e Time-Slotted Channel Hopping (TSCH) networks should be equipped to deal with the hard wireless challenges of industrial environments, the sensor networks are often still limited by the characteristics of the used physical (PHY) layer. Therefore, the TSCH community has recently started shifting research efforts to the support of multiple PHY layers, to overcome this limitation. On the one hand, integrating such multi-PHY support implies dealing with the PHY characteristics to fit the resource allocation in the TSCH schedule, and on the other hand, defining policies on how to select the appropriate PHY for each network link. As such, first a heuristic is proposed that is a step towards a distributed PHY and parent selection mechanism for slot bonding multi-PHY TSCH sensor networks. Additionally, a proposal on how this heuristic can be implemented in the IPv6 over the TSCH mode of IEEE 802.15.4e (6TiSCH) protocol stack and its Routing Protocol for Low-power and Lossy network (RPL) layer is also presented. Slot bonding allows the creation of different-sized bonded slots with a duration adapted to the data rate of each chosen PHY. Afterwards, a TSCH slot bonding implementation is proposed in the latest version of the Contiki-NG Industrial Internet of Things (IIoT) operating system. Subsequently, via extensive simulation results, and by deploying the slot bonding implementation on a real sensor node testbed, it is shown that the computationally efficient parent and PHY selection mechanism approximates the packet delivery ratio (PDR) results of a near-optimal, but computationally complex, centralized scheduler.

## 1. Introduction

Industrial applications that are deployed on low-power wireless sensor networks often demand a trade-off between data rate, energy consumption, latency and reliability. To fit the exact requirements, IPv6 over the TSCH mode of IEEE 802.15.4e 6TiSCH networks allow the tuning of the network characteristics at different layers of the network stack, starting from the IEEE 802.15.4e Time-Slotted Channel Hopping (TSCH) Medium Access Control (MAC) layer up to the application layer [1,2]. TSCH was introduced to combat external interference and multi-path propagation effects in challenging wireless sensor network environments. To do so, it uses a tightly synchronized time-slotted schedule and pseudo-random channel hopping to provide high reliability while maintaining low-power operation. Nevertheless, the 6TiSCH Industrial Internet of Things (IIoT) stack is still limited by the characteristics of the chosen physical (PHY) layer, which is often challenged by the harsh conditions of industrial environments filled with other wireless devices and metal obstacles. To overcome this limitation, the TSCH and 6TiSCH community is shifting research efforts to the support of multiple PHY layers [3,4,5,6].

Employing different PHYs in a single TSCH network also means embracing the different characteristics of each PHY, such as its data rate, bandwidth, computational complexity, energy consumption and wireless reliability. Integrating multi-PHY support in a 6TiSCH sensor network therefore implies, on the one hand, dealing with the different transmission lengths and bandwidth requirements to fit the PHY allocation in the TSCH schedule and, on the other hand, defining policies on how to select the appropriate PHY for each network link. In our previous work, the question of how different PHYs can be fitted into a TSCH schedule was answered by introducing the concepts of slot and channel bonding [7]. The former allows the combination of multiple TSCH time slots of regular length into a larger bonded slot. The length of the resulting bonded slot is large enough to transmit or receive a packet using the data rate of the PHY selected for the link. The concept of the latter is similar, but now multiple frequency channels are bonded together to a channel wide enough to communicate using the selected PHY. Slot and channel bonding are considered resource-efficient as both adapt the resources (i.e., the time slots and channels) exactly to the requirements of the selected PHYs. In this work, to facilitate the simultaneous use of multiple PHYs in a single TSCH sensor network, the slot bonding technique is used. In particular, the parent and PHY selection for each network node is investigated, related to making correct routing decisions based on the available PHYs and being able to schedule those PHY resources in the TSCH schedule.

The contributions of this article are two-fold. First, a computationally efficient heuristic is proposed that is a step towards a distributed PHY and parent selection mechanism for a TSCH network that applies slot bonding. The performance of this heuristic is initially evaluated via simulation results, by comparing it against a near-optimal, but computationally complex, multi-PHY scheduler. Additionally, a proposal on how this heuristic can be implemented in the Routing Protocol for Low-power and Lossy network (RPL) is also presented. Second, an easy-to-configure TSCH slot bonding implementation is proposed in the latest version of the Contiki-NG IIoT operating system. Subsequently, this slot bonding implementation is used to extensively evaluate the proposed heuristic on a real hardware sensor testbed.

The remainder of this article is structured as follows: first, the related work on using multi-PHY TSCH is discussed in Section 2. Second, in Section 3, the parent and PHY selection heuristic is introduced. In Section 4, the TSCH slot bonding implementation in the Contiki-NG firmware is described. Afterwards, the heuristic is evaluated in Section 5. Finally, Section 6 presents the conclusions of this work and in Section 7, an overview of the future work is provided.

## 2. Related Work

The introduction of the IEEE 802.15.4g amendment and the commercial availability of IEEE 802.15.4g-compliant transceivers has spiked research on alternative PHYs and their potential for 6TiSCH networks. There are many works carrying out experimental evaluations including range tests, interference robustness and the overall suitability of different IEEE 802.15.4g PHYs, tested outdoors, in smart buildings or in industrial scenarios [8,9,10,11,12,13,14].

In our previous work, the slot bonding technique was proposed to combine multiple PHYs with various data rates in a single TSCH network. It allows the combining of multiple TSCH time slots of regular length into a bonded slot with a length large enough to transmit or receive a packet using the data rate of the PHY selected for the link [7]. By adapting the length of the bonded slot as closely as possible to the duration required by the data rate of the selected PHY, slot bonding avoids wasting valuable airtime. Subsequently, the TSCH slot problem was described formally in terms of optimizing the packet delivery ratio while keeping the radio on time to a minimum. To be able to solve this slot bonding problem, a Genetic Algorithm (GA) was introduced. The GA could find the parent, PHY and number of slot selections in a multi-PHY slot bonding TSCH network in a near-optimal manner. As this GA was computationally too complex to be used in a real-time TSCH network, in this work, a computationally efficient parent and PHY heuristic is proposed.

Currently, research that investigates specifically how different PHYs can be combined in a single TSCH network, is scarce. Rady et al. adjusted the OpenWSN firmware to support Offset-Quadrature Phase Shift Keying (O-QPSK) (2.4 GHz at 250 kbps), Frequency Shift Keying (FSK) ( 868 option 1 at 50 kbps) and Orthogonal Frequency Division Multiplexing (OFDM) (868 MHz option 1 MCS3 at 800 kbps) [6]. For a fair comparison, the authors used a 40 ms time slot that could facilitate all implemented PHYs. After evaluating the different PHYs on a 42 node office testbed, the authors conclude that no PHY outperforms the other PHYs for all metrics, and therefore the combination of different PHYs should be considered on a frame-by-frame basis (depending on the changing propagation characteristics of the link) in a 6TiSCH architecture. Defining a policy on how to select the appropriate PHY was not in the scope of the article. In later work, Rady et al. proposed 6DYN, an extension of the 6TiSCH stack with heterogeneous slot durations for different PHYs, implemented in OpenWSN [15]. The authors provide three different minimal cells in the TSCH schedule, one for each employed PHY. Additionally, they provide a separate row in the neighbor table for each different PHY of the same neighbor. These decisions allow for a seamless integration of the PHY and MAC layers in the routing layer and could be merged with the parent and PHY selection mechanism proposed in this work. 6DYN is evaluated in a rather small experiment setup of four motes as a more elaborate evaluation is kept for future work. Gomes et al. propose different policies on packet replication and the use of multiple IEEE 802.15.4g modulations for different packets, that is, modulation diversity, to increase network reliability [16]. While how to exactly integrate the different modulations in TSCH is not discussed, the proposed policies should be considered as PHY selection mechanisms that can be applied on top of our slot bonding approach. Another recent interesting approach is presented by Van Leemput et al., who take into account the different data rates possible for different PHYs [4]. The authors defined two alternative timeslot structures allowing multiple packet transmissions to increase the throughput for higher data rate PHYs while maintaining a fixed slot duration. This approach is different from the slot bonding, in which only one packet is transmitted, but the slot length is adapted to the chosen PHY [7]. Additionally, they developed a flexible Link Quality Estimation technique to dynamically switch between PHYs depending on the current propagation characteristics of the link. Another interesting work is the approach of Brachmann et al. They proposed two multi-PHY designs: (i) a *multi-template* design where slower PHYs are scheduled to have logical slots spanning multiple real slots, and (ii) a *single-template* design where the slot size is based on the slowest PHY [3]. Their multi-template design is similar to the slot bonding approach presented in our previous work. The real hardware evaluation of Brachmann et al. applies their multi-template design by assigning management traffic to the slower, more reliable PHY, while data are transmitted over the faster, less reliable PHY. As such, they do not not discuss a PHY selection mechanism and their results primarily focus on the advantages of using a multi-PHY in terms of range, channel utilization and synchronization accuracy. The TSCH slot bonding firmware implementation presented in this work is a combination of a new easy-to-configure implementation, developed in the latest version of the Contiki-NG repository, merged with insights from the open-source multi-PHY work from both Van Leemput et al. and Brachmann et al.

To the best of our knowledge, this work is the first to evaluate a parent and PHY selecting mechanism in a TSCH slot bonding, multi-hop network deployed on a real hardware testbed.

## 3. Parent and PHY Selection Heuristic

In this section, a computationally efficient heuristic algorithm is proposed that allows TSCH slot bonding nodes to make a parent and PHY selection. The heuristic is a step towards a distributed selection mechanism that could be integrated into the RPL or any other routing protocol. First, it is re-iterated how parent nodes are normally selected in a single PHY network and afterwards the motivation for this heuristic is explained. Then, the heuristic itself is presented. As a conclusion, insight into how this heuristic could be integrated into RPL is provided.

### 3.1. IEEE 802.15.4e TSCH

In an IEEE 802.15.4e TSCH network, each node maintains a matrix-formatted schedule, in which the number of columns represents the number of time slots within one slotframe [17]. This slotframe is repeated over time and its size is typically application-dependent. The number of rows of the schedule represents the number of available physical channels. One element in the schedule is called a cell and is denoted by (*timeOffset*, *channelOffset*) with, respectively, the time and channel offset in the schedule. As such, each node is time-synchronized, with time being split up into fixed-duration time slots, which are indexed by the time offset and at every slot a node can use one of the available channels, which is calculated based on the channel offset. A default time slot is typically long enough to send a packet of 127 bytes and receive an acknowledgement (ACK), using the predetermined physical layer applied by all the nodes in the network. To define absolute time, the TSCH MAC layer uses the so-called Absolute Sequence Number (ASN), which is 0 at the very first time slot and is incremented by one at every time slot that follows.

By telling a node exactly what to do in each slot, the TSCH MAC layer is capable of very low-power operation. The different possible actions of each slot are transmitting, receiving or sleeping. When a scheduled cell is marked for transmission, the node will transmit the first packet in the queue. In a receiving scheduled cell, a node listens for an incoming packet. A *dedicated* cell between two nodes is a cell reserved only for those two nodes. When more nodes are allowed to use a cell for transmission, this cell is called a *shared* cell in which the collision probability is kept to a minimum by using a back-off algorithm. To bootstrap the TSCH network and allow all nodes to disseminate network and routing information across the network, each TSCH schedule typically has a so-called *minimal shared cell* [18]. To achieve very reliable communication, TSCH applies channel hopping (which helps to avoid external interference and destructive multi-path fading effects) by taking into account the ASN of the cell together with its channel offset, to select the frequency channel of the transmission in a pseudo-random manner.

**Figure 1:**
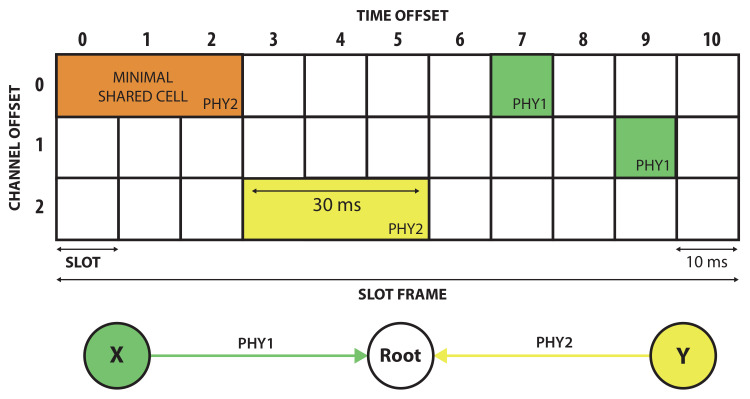
A TSCH schedule with regular slots of 10 ms length. Node X uses physical layer PHY1 that only requires one regular time slot of 10 ms. For accommodating physical layer PHY2 on the link from node Y to the root, three slots are bonded together to a 30 ms slot. For the minimal shared cell, the most robust PHY2 is used.

Figure 1 shows an example of a TSCH schedule. More specifically, node X illustrates a traditional TSCH reservation of two regular time slots for its data transmissions to the root node. Using PHY1, the transmission of the frame and the reception of the acknowledgment fits in one time slot of 10 ms.

### 3.2. Slot Bonding

While, traditionally, TSCH only uses a single PHY per network, slot bonding allows the combination of multiple PHYs in one single TSCH network. As such, the performance of each node in the network can significantly improve by adapting its PHY to the local propagation characteristics and the application’s requirements.

To combine different PHYs in a single TSCH schedule, slot bonding takes into account the data rates of the different available PHYs. More specifically, for a specific PHY, it bonds multiple time slots of *regular* length together to a *bonded* slot that is long enough to fit the transmission of a data packet and the reception of an acknowledgement. As such, by adapting the length of each bonded slot to the data rate of the used PHYs, slot bonding can combine different PHYs in a resource-efficient manner. In contrast, as explained earlier, traditional TSCH relies on fixed-duration slots, long enough to send a packet of any size given the fixed data rate, which results in wasted airtime if different PHYs with different data rates are used simultaneously. In our previous work, it was shown that the resource-efficiency of slot bonding results in a better network overall packet delivery ratio [7]. For more results and a detailed explanation on slot bonding, the reader is referred back to that work.

Figure 1 shows that node Y needs PHY2 to send its data to the root node. Therefore, it needs to bond three regular time slots together to one bonded slot of 30 ms. Similarly, the minimal shared cell is also configured with PHY2, as this is considered the PHY with the best robustness and range features, allowing all nodes to bootstrap and receive network and routing information.

### 3.3. RPL Parent Selection

In 6TiSCH networks, it is the RPL routing protocol that organizes the network paths along which data are transmitted from the originating node to the network sink [19]. RPL organizes the network nodes in a tree topology, which is rooted at the sink node (i.e., a border router). Each node in the RPL routing tree can have zero or more child(ren), and one or more parent(s). A node that has no children is a leaf node, and the node in the tree that does not have any parents is the root node of the tree. When data have to be transmitted to the sink (or root) of the network, they are forwarded on the link between every node and its parent along the path from the originating node towards the root. The location of the node in this routing tree is determined by its *Rank* value. The node calculates this value itself and broadcasts this information in so-called Destination Oriented Directed Acyclic Graph Information Object (DIO) messages. To calculate this Rank value, but also to select the preferred routing parent, a node applies an Objective Function (OF) that uses one or more routing metrics to approximate the distance of the node to the root. The minimal 6TiSCH profile mandates using the default Objective Function Zero (OF0) [18,20]. More specifically, when a node applies OF0 to select a parent among its set of neighbors, it uses a so-called *step_of_rank* value, which represents the link properties to the specific neighbor. That *step_of_rank* becomes part of a normalized *rank_increase* value that is added to the Rank value received via routing broadcast messages (i.e., DIO messages) from each neighbor. The neighbor with the lowest resulting Rank value is picked as the (new) preferred parent (only if the difference with the rank of the previous parent exceeds a parent switching threshold). The minimal 6TiSCH profile defines this *step_of_rank* as a function of the Expected Transmission Count (ETX) (i.e., the estimated number of needed transmission attempts for one successful transmission) towards that neighbor. An alternative to OF0 is the Minimum Rank with Hysteresis Objective Function (MRHOF) [21]. Using hysteresis to limit the number of topology changes caused by only small metric changes, this Objective Function (OF) selects routes that minimize an additive metric. Those metrics are usually advertised in the so-called Metric Containers of RPL DIO messages. When no metrics are being advertised in DIO messages, the OF also uses the ETX metric to make parent selections.

### 3.4. Motivation

In our previous work, a GA was defined that acted as a centralized scheduler for multi-PHY TSCH networks by making parent, PHY and slots selections for each node in the network [7]. This GA allowed us to implement the slot bonding problem and find solutions in near-optimal fashion. As such, it was used for the analysis of the proposed multi-PHY TSCH slot bonding technique. Due to its time complexity and its centralized approach, it is not feasible to use it for real-time parent and PHY selections in dynamic TSCH networks.

Therefore, in this work, a computationally efficient heuristic is presented that allows nodes to make parent and PHY selections in a multi-PHY 6TiSCH network. The algorithm is a step towards a distributed approach that can be integrated in RPL OFs, taking into account the ETX and a PHY characteristic, that is, the data rate that is translated into the number of slots needed to bond together to transmit a packet.

### 3.5. Heuristic

The proposed heuristic aims to optimize the overall packet delivery ratio (PDR) of the network. Its inputs are a reliability threshold value (i.e., δ, which defines the minimum reliability a selected PHY for a parent node should have, compared with the most reliable PHY to that parent node), the data rates of the available PHYs and, for each node, the link reliability values for all PHYs to all the node’s possible parents. The output of the heuristic is a preferred parent and a PHY, to connect to that parent, for each node in the network. To achieve its goal, the heuristic first selects, for each node, a preferred PHY for each of its possible parents. Afterwards, taking into account those PHYs for each possible parent, the heuristic minimizes the number of allocated slots from each node to the root. Therefore, it considers the bonded slot length necessary for each PHY and the link reliability to that possible preferred parent. Both steps of the heuristic are described in Algorithms 1 and 2 and are discussed in detail below.
**Algorithm 1.** Procedure to determine the preferred PHY per possible parent.1: **procedure**
mapPhyPerParent(*n*, δ)2:      $m_{n}=map\left(\right)$                              ▹ for node *n*, map possible parents to PHY3:      **for** p $\in P_{n}$**do**                     ▹ loop over every possible parent of node *n*, in $P_{n}$4:          $m_{reliable}=None$                                   ▹ most reliable PHY to parent *p*
5:          **for** m $\in M_{p}$
**do**                                                 ▹ find most reliable PHY
6:               **if**
$reliability\left(m\right)>reliability\left(m_{reliable}\right)$
**then**
7:                    $m_{reliable}=m$            $m_{n}\left[p\right]=m_{reliable}$
8:          **for** m $\in M_{p}$
**do**                                               ▹ find possible faster PHY
9:               **if**
$rate\left(m\right)>rate(m_{n}\left[p\right])$
**then**
10:                  **if**
$reliability\left(m_{reliable}\right)-reliability\left(m\right)\leq \delta$
**then**
11:                       $m_{n}\left[p\right]=m$
12:      **return**
$m_{n}$


In the first step of the heuristic, each node in the network, except the root (i.e., each node *n* in the set $N_{0}$), assigns a preferred PHY to each possible parent in its set of possible parent nodes. To do so, it considers the trade-off between reliable and/or fast PHYs, as it was observed in the allocation analysis of our previous work that both metrics are important when picking a parent [7]. This PHY selection procedure is shown in Algorithm 1 (and this procedure is in Algorithm 2 at line 3). Given node *n*, this algorithm first searches for every possible parent of *n* (i.e., in set $P_{n}$), for the most reliable PHY, $m_{reliable}$ from node *n* to that parent *p*. Afterwards, based on the known data rate of each possible PHY, the fastest PHY is selected that is within a given threshold (i.e., the parameter δ) of the most reliable PHY to that parent. Formally, this means that for the selected PHY *m* to a possible parent the link reliability $l_{m}$ is such that $l_{m}\geq l_{max}-\delta$ with $l_{max}$ being the highest possible reliability among all PHYs supported by the node to that possible parent. Choosing a small δ value will lead to more reliable links between a node and its parent, but also leads to less free space in the schedule as PHYs with lower data rates need more time to transmit a packet and thus need more regular slots to be bonded together. Choosing a large δ allows less reliable PHYs to be chosen, and assuming that less reliable PHYs have faster date rates that need a shorter bonded slot to transmit a packet (this assumption is largely confirmed by the PDR results of the different IEEE 802.15.4g modulations in [8]), this will lead to more free space in the schedule. Specifically, if δ=0, the heuristic always chooses the most reliable PHY for each possible parent, while when δ=1, the heuristic always chooses the PHY with the fastest data rate. The value of δ should thus be determined empirically and can be network-, application- or even node-specific.
**Algorithm 2.** Heuristic to find preferred parents and PHYs.1: **procedure** assignParentAndPhy(α, δ)
2:      **for**
$n\in N_{0}$
**do**                  ▹ for every *n*, assign the preferred PHY per parent
3:          $m_{n}$ = mapPhyPerParent(n, δ)
4:      converged = false
5:      **while** ¬ converged **do**           ▹ stop when no node changed parent anymore
6:          converged = true
7:          $x_{0}=0$                                                     ▹ score of root initialized to 0
8:          **for**
$n\in N_{0}$
**do**
9:               $p_{n}=None$                                          ▹ preferred parent of node *n*
10:              $x_{n}=None$                                     ▹ score to preferred parent of *n*
11:              **for**
$p\in P_{n}$
**do**
12:                   **if**
$x_{p}\neq None$
**then**                  ▹ only if possible parent has a score
13:                         $ETX=\frac{1}{reliability(m_{n}\left[p\right])}$                             ▹$ETX=\frac{1}{reliability}$
14:                          $x=x_{p}+ETX\;\cdot \;s_{m_{n}\left[p\right]}$  ▹$s_{m}$ is bonded slot length of PHY *m*
15:                          **if**
$x_{n}=None\vee x<x_{n}$
**then**
16:                                $x_{n}=x$
17:                                $p_{n}=p$
18:                                converged = false                           ▹ there was a change
19:      **return**
$\left\{p_{0},p_{1},\ldots ,p_{|N_{0}|}\right\},\left\{m_{0}\left[p_{0}\right],m_{1}\left[p_{1}\right],\ldots ,m_{|N_{0}|}\left[p_{|N_{0}|}\right]\right\}$


Having selected a PHY for each possible parent for each node *n*, from line 5 onward in Algorithm 2, the heuristic searches for the preferred parent $p_{n}$ of every node *n*. The heuristic stops when every node has selected a preferred parent. Every node *n* is assigned a so-called *score* $x_{n}$ and the score of the root is 0, assigned at line 7. The algorithm tries to minimize this score for each node as it represents the expected number of regular slots (i.e., not *bonded* slots) needed to transmit a packet along the path from the node to the root. For every node *n*, it loops over every possible parent *p* of node *n* and, if that parent was already assigned a score $x_{p}$, it calculates a new score *x* for node *n*. On line 13, this score is calculated by using the ETX value for the PHY selected for that possible parent. Afterwards, on line 14, that ETX is multiplied with the bonded slot length of that PHY (i.e., $s_{m}$, the number of regular slots that need to be bonded together to transmit a packet, using PHY *m*), and added to the score $x_{p}$ of the parent. If it is the first time the score is assigned to the node *n* or the score is smaller than the previous $x_{n}$, this possible parent becomes the new preferred parent (see lines 15–17). By using the ETX and the bonded slot length for the score calculation, and by looking for the smallest parent score $x_{p}$, the heuristic actually aims to pick a preferred parent with a path to the root that minimizes the number of allocations in the schedule. It was observed in the results of our previous work that more free space leads to higher PDR values; this way the heuristic tries to optimize the space in the schedule so every node can make sufficient allocations to successfully (re-)transmit their packets [7]. On line 18, it is denoted that there was a change in topology as this may affect other scores of other nodes. As such, the heuristic should not converge yet and keep running until no changes happen. Finally, when converged, the heuristic returns the sets of chosen parents and PHYs for each node.

**Figure 2:**
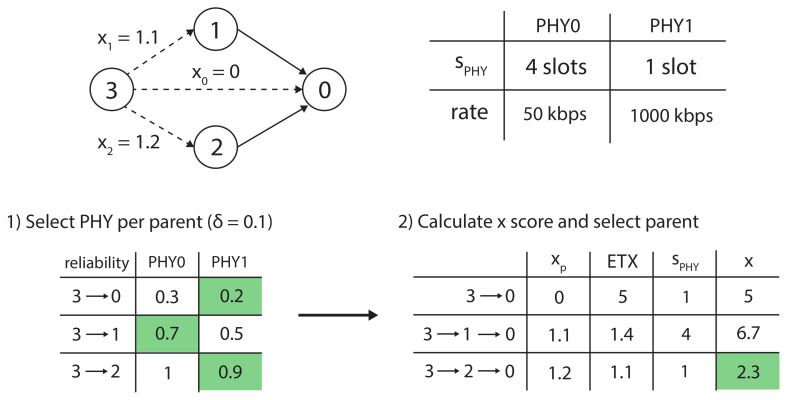
Example of node 3 using the heuristic to select its parent and PHY. First, the node selects the PHY per parent (with threshold δ=0.1) and afterwards the node calculates the *x* score to select parent 2.

An example of the heuristic is given in Figure 2. Node 3 uses the heuristic to choose a preferred parent between nodes 0, 1 or 2 and a PHY to that parent. First, it selects a PHY per parent, with δ=0.1. Afterwards, it calculates the *x* score using the score from each possible parent, $x_{p}$, the ETX value to that parent and the bonded slot length for the preferred PHY to that parent, $s_{PHY}$, to select node 2 as its new preferred parent (using preferred PHY PHY1).

The total time complexity of the proposed heuristic can be analysed by examining the time complexity of the two parts of the heuristic in Algorithm 2, that is, from line 2 to line 4 (including Algorithm 1) and from line 5 to line 18. For the former, the time complexity is O(|N|·|P|·|M|) (with *N*, *P* and *M* being the total set of nodes, the set of possible parents and the set of available PHYs respectively), while for the latter, the time complexity is O(c·|N|·|P|), where *c* represents the maximum number of iterations needed to achieve convergence (i.e., the while loop at line 5). Therefore, the total time complexity of the heuristic is $O(c\;\cdot \;|N{|}^{2}\;\cdot \;|M|)$, as |P|≤|N|. To limit the influence of c on the computational complexity of the algorithm, a maximum bound can be set on the number of allowed iterations. Our experiments (cf., Section 5) show that the number of iterations needed for convergence are consistently below five (with an average of 3.5 iterations) in the evaluated scenarios. This analysis proves the computational efficiency of the heuristic compared to the time complexity of the centralized GA scheduler, that is, $O(|N|!\;\cdot \;|N{|}^{2})$, as explained in detail in our previous work [7].

### 3.6. RPL Integration

While the proposed heuristic is defined from a centralized point of view (and it can be used as a centralized scheduler), it is clearly a step towards a distributed parent and PHY selection mechanism. Here, an explanation is provided of how the different inputs of the heuristic can be obtained and how the heuristic can be integrated into an existing low-power routing protocol, that is, the RPL routing protocol, part of the 6TiSCH stack. More specifically, how the heuristic can be integrated into the RPL OF in a distributed way (i.e., utilizing only information that is locally available at each node) is discussed.

The inputs of the heuristic are the data rates of the different PHYs, the different link reliability values (or $ETX=\frac{1}{reliability}$) and the score values of possible parent nodes. The data rates of the different PHYs and the related number of regular slots that need to be bonded together to transmit a packet, $s_{m}$, are known by every node beforehand as they are characteristic for each PHY. The different reliability values could be approximated by mapping neighbor Received Signal Strength Indicator (RSSI) values to PDR values, based on PHY measurements carried out in the environment where the network is being deployed. For example, Contiki-NG (https://github.com/contiki-ng/contiki-ng/blob/develop/os/net/link-stats.c, accessed on 12 May 2021) has the option called LINK_STATS_INIT_ETX_FROM_RSSI to estimate the ETX from RSSI values [22]. This could be extended to support multiple PHYs. Alternatively, it is also possible to learn the link reliability over time. The link reliability can be defined as the number of ACKs over the number of transmission attempts, which can then be turned into an $ETX=\frac{num\_tx}{num\_ack}$, as it is done in Contiki-NG (i.e., option LINK_STATS_ETX_FROM_PACKET_COUNT) but also in the 6TiSCH OpenWSN implementation (https://github.com/openwsn-berkeley/openwsn-fw/blob/develop/openstack/02b-MAChigh/neighbors.c, accessed on 12 May 2021). When no transmission attempts are available for a certain possible parent, a default ETX value can be chosen (e.g., representing an average link quality). In this case, where link reliability is learned over time and a node has to find a fast and reliable PHY for the current preferred parent (as proposed in Algorithm 1), a node can apply a Link Quality Estimation (LQE) technique as proposed by Van Leemput et al. to iterate through the different PHYs [4]. The necessary score values $x_{p}$ of possible parents can be distributed throughout the network using the Metric Container option in RPL DIO messages, similar to the metrics distributed for the MRHOF OF [19,21].

Using those inputs, the MRHOF OF can be used to further integrate the heuristic, as that OF is designed to minimize the path cost based on additive metrics (that are turned into Rank values), that is, the score values used in the heuristic. Subsequently, using MRHOF, the node will select (with hysteresis) a parent that requires fewer defaults slots to be scheduled along the path towards the root, as this is the goal of the proposed heuristic.

**Figure 3:**
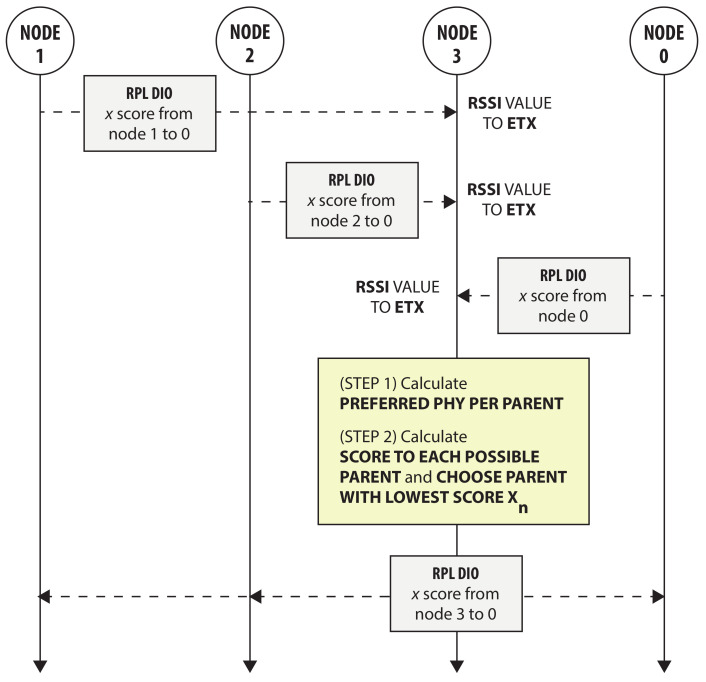
Illustration of the distributed implementation of the heuristic in the RPL routing layer. Node 3 uses the heuristic to select a new PHY and parent, by using the received *x* scores to the root node 0 and the new reliability values (based on the calculated RSSI values). After calculating the lowest possible *x* score from node 3 to node 0 (and thus also selecting its preferred parent and PHY), this score is broadcast throughout the network. Normally, all RPL DIO messages are broadcast to all nodes, but this is omitted for node 0, 1 and 2 to make the figure clear.

Figure 3 illustrates a simplified example of how the heuristic can be integrated in a distributed fashion in the RPL routing layer. The figure uses the same scenario and nodes as in Figure 2 in which node 3 uses the heuristic to select a new parent and PHY. First, node 3 receives RPL DIO messages from each node containing the *x* score (in the Metric Container of the DIO message) from each originating node to node 0 (i.e., the root node). Normally, these RPL DIO messages are broadcast to all nodes, but this is omitted in order to make the figure clear. When receiving the DIO messages, node 3 determines the RSSI value of each link and subsequently calculates the reliability for each PHY per possible parent as defined in Algorithm 1 (and it is assumed in this example that an option like LINK_STATS_INIT_ETX_FROM_RSSI of the Contiki-NG firmware is used and implemented for each possible PHY). In the next step, node 3 calculates the lowest possible score, $x_{n}$, using the new reliability values and the bonded slot lengths of the preferred PHY per possible parent, and as such selects a new preferred parent. Finally, node 3 broadcasts this new score to its neighbors. Subsequently, the new score originating from node 3 can influence the parent and PHY choice at the neighboring nodes and the same process can take place at each of the neighbors.

## 4. Slot Bonding Implementation

This section provides an overview of our proof-of-concept TSCH slot bonding implementation in the Contiki-NG firmware. The implementation is publicly available (https://github.com/imec-idlab/tsch-slotbonding, accessed on 12 May 2021). First, the development platform is discussed. Second, the different PHYs that were considered and the possible channel allocations are explained. Finally, the TSCH timing values used in the implementation are discussed.

### 4.1. Platform

The slot bonding proof-of-concept is implemented in the latest version (https://github.com/contiki-ng/contiki-ng, commit 5fdfc98, accessed on 12 May 2021) of the Contiki-NG firmware [22]. Contiki-NG is an open-source, cross-platform operating system for Internet of Things (IoT) devices with support for the 6TiSCH stack. The implementation is developed on the Zolertia hardware platform, more specifically the RE-Mote (revision B) platform featuring the Texas Instruments CC1200 sub-GHz low-power transceiver (http://www.ti.com/product/CC1200/datasheet/, accessed on 9 May 2021). As the Contiki-NG firmware is ported to many different platforms, the slot bonding implementation can also be used on different hardware.

### 4.2. PHYs

The implementation supports the 2 sub-GHz PHY configurations shown in Table 1, that is, the 2-GFSK modulation with 200 kHz channel spacing and 50 kbps data rate and the 4-GFSK modulation with 1667 kHz channel spacing and 1000 kbps data rate. PHY layers in the sub-GHz spectrum are used because of the better propagation characteristics compared to the 2.4 GHz spectrum [23]. Both CC1200 radio configurations are by default available in the Contiki-NG repository. The implementation can be extended to support more PHY radio configurations. As indicated in the work of Van Leemput et al. and as seen in Table 2, taking into account the strict regulation of the sub-GHz spectrum, 44 channels of 200 kHz and 4 channels of 1667 kHz are possible (with frequency overlap) in the 7 sub-GHz bands divided over the European Union 863-870 MHz and 873-920 MHz spectra [4,24]. Such a channel allocation with frequency overlap actually avoids wasting bandwidth and allows for a rich frequency diversity. However, the frequency overlap can also result in inter-PHY interference and possible duty cycle saturation for certain frequency bands. The alternative is allocating channels for the different PHYs without frequency overlap (thus reserving different parts of the wireless spectrum for different PHYs), with the consequence of limiting the frequency diversity.

### 4.3. Timing Values

TSCH slot bonding bonds multiple *regular* time slots into a bonded slot that is long enough to transmit or receive a frame of 128 bytes (125 data bytes, 2 CRC bytes and 1 byte frame length), given the data rate of the selected PHY [7]. As such, each bonded slot is tailored to the requirements of the specific PHY and thus has the advantage of limiting the waste of airtime resources.

**Figure 4:**
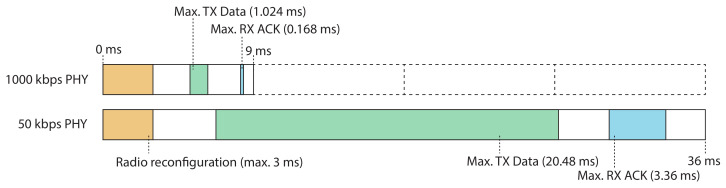
Illustration of the slot timings for the 1000 kbps and 50 kbps (bonded) slots.

As described in Section 4.2, the two available CC1200 PHYs with data rates of 50 kbps and 1000 kbps are used. The differences between the two PHYs and their data rates are reflected in the total time slot lengths and the timing values for the different states within a time slot, as defined in the radio configurations of these PHYs. Brachmann et al. list these different timing values and how they can be determined [3]. The largest difference between the two PHYs is reflected in the transmission length of the data frame due to the difference in data rate (i.e., in kbps), as the transmission length equals $\frac{128\times 8}{datarate}$ milliseconds. Using the timings as defined in their original radio configurations, the total time slot lengths are 31.46 ms and 5.808 ms for 50 kbps and for 1000 kbps, respectively. However, the extra time at the start of the slot to reconfigure the radio for the appropriate PHY also has to be included. Similar to Brachmann et al., 3 ms for this reconfiguration per time slot was added, bringing the total time slot lengths to 34.46 ms and 8.808 ms. As slot bonding requires bonding multiple slots together to form a longer time slot for PHYs with slower data rates, the total time slot length of the fastest PHY should be a common divisor of the time slot lengths of the slower PHY(s). Therefore, the slack time at the end of both time slots was extended to end up with total lengths of 9 ms and 36 ms, as shown in Table 1. This means that the *regular* time slots in the TSCH schedule have a length of 9 ms (meaning that the TSCH ASN is incremented every 9 ms) and are used by the 1000 kbps PHY. When allocating a slot for the 50 kbps PHY, four regular slots of 9 ms will be bonded together. Figure 4 illustrates the differences between the time slots of the two chosen PHYs.

## 5. Evaluation

In this section, the proposed parent and PHY selection heuristic is evaluated. First, our experiment setup and methodology are presented. Afterwards, the 6TiSCH simulator is used to compare the heuristic against the near-optimal, computationally complex GA approach [7]. Finally, the performance of the heuristic running on a real hardware testbed is evaluated using the proposed slot bonding implementation.

### 5.1. Experiment Methodology & Setup

First, the experiment methodology and setup for the simulation experiments are described, afterwards the testbed experiments are discussed.

#### 5.1.1. Simulator Experiments

In the simulation experiments, a comparison was carried out between the proposed parent and PHY heuristic of Section 3 and the near-optimal, but computationally complex, GA scheduler that was introduced in our previous work [7]. The experiment workflow using the GA consisted of four consecutive steps. In the first step, the 6TiSCH simulator was used to generate the network topologies [25]. Then, in the second step, these topologies were used as input to the GA. While optimizing the packet delivery ratio and keeping the radio on time to a minimum, the GA solved the slot bonding problem, based on the generated topologies, in a near-optimal manner. The simulator and the GA are publicly available (https://github.com/imec-idlab/tsch-slotbonding-ga-simulator, accessed on 8 May 2021). During the third step, our so-called feasibility algorithm generated an overall TSCH schedule for all network nodes, using the best possible solution found by the GA (including the parent and PHY selection and the number of slots for each node). In the fourth and last step, the resulting schedule was used in the 6TiSCH simulator as a centralized schedule to make static schedule allocations in the TSCH experiments. For a more elaborate description and illustration of this experiment setup, the reader is referred back to our previous work [7].

The proposed heuristic only makes parent and PHY selections and does not allocate (bonded) slots. Therefore, using the network topology created by the 6TiSCH simulator in the first step of the experiment process, the proposed heuristic made the parent and PHY selections and fixed these selections, and afterwards the GA was configured to find the best slot allocation solution for those parent and PHY choices. The reason for keeping the GA scheduler for the slot allocations was to have a fair comparison for the PHY and parent selection, without having influence from (sub-optimal) slot allocation. This implementation is publicly available (https://github.com/imec-idlab/tsch-slotbonding, accessed on 8 May 2021).

#### 5.1.2. Testbed Experiments

**Figure 5:**
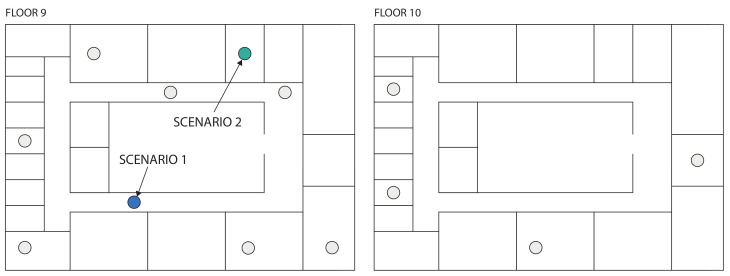
The two floors of the hardware testbed with the 13 used nodes illustrated at their locations. Two scenarios of 12 nodes were considered: scenario 1 includes the 11 grey nodes and the blue node while scenario 2 includes the 11 grey nodes and the green node.

**Figure 6:**
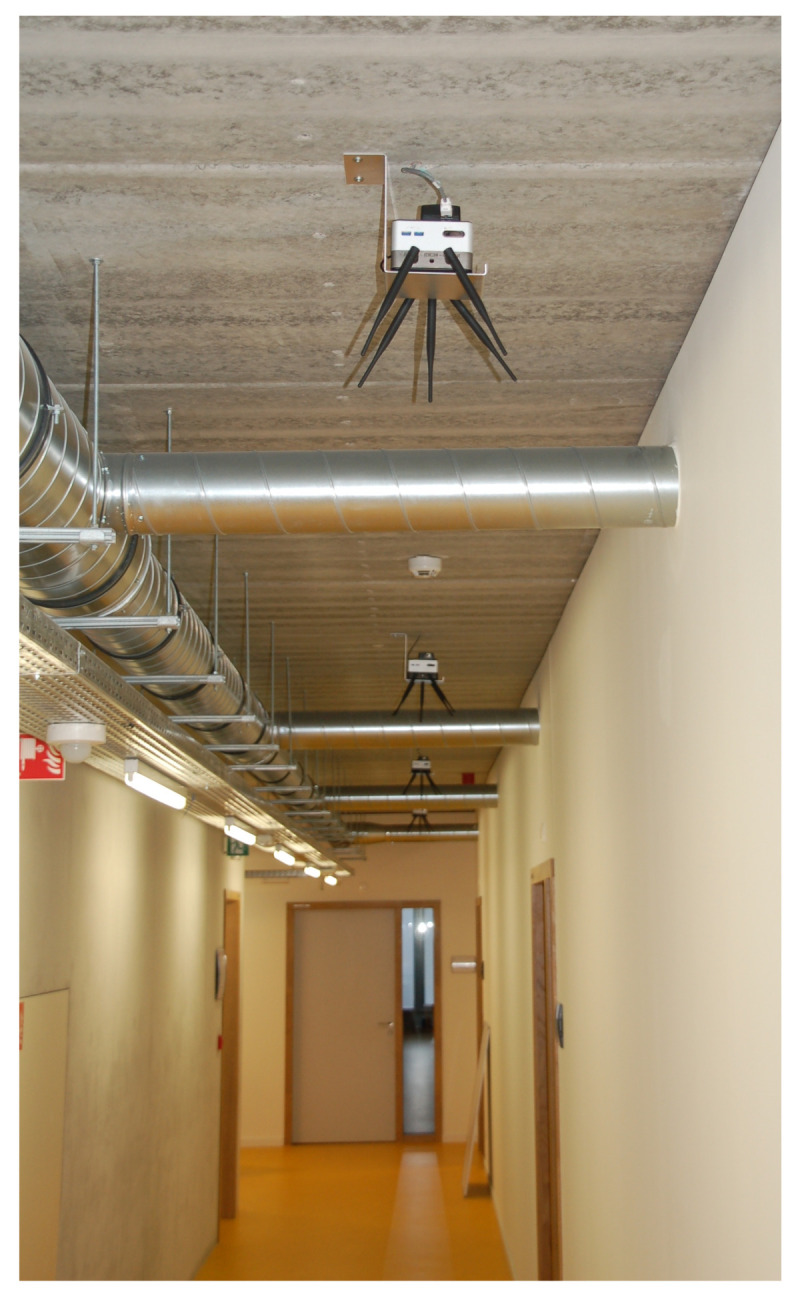
A subset of *imec Wireless OfficeLab* sensor nodes, deployed on the ceiling in an office environment spanning different floors of 805 m${}^{2}$, separated by reinforced concrete.

The proposed heuristic was also validated on the *imec Wireless OfficeLab*, using the TSCH slot bonding proof-of-concept discussed in Section 4 [26]. The testbed was located in an office environment spanning different floors of 805 m${}^{2}$ separated by reinforced concrete, with a total of 150 sensor nodes of which a subset of 13 nodes was used as illustrated in Figure 5. The deployed sensor nodes were the Zolertia RE-motes [27]. Figure 6 shows several of the deployed sensor nodes in the office environment.

**Figure 7:**
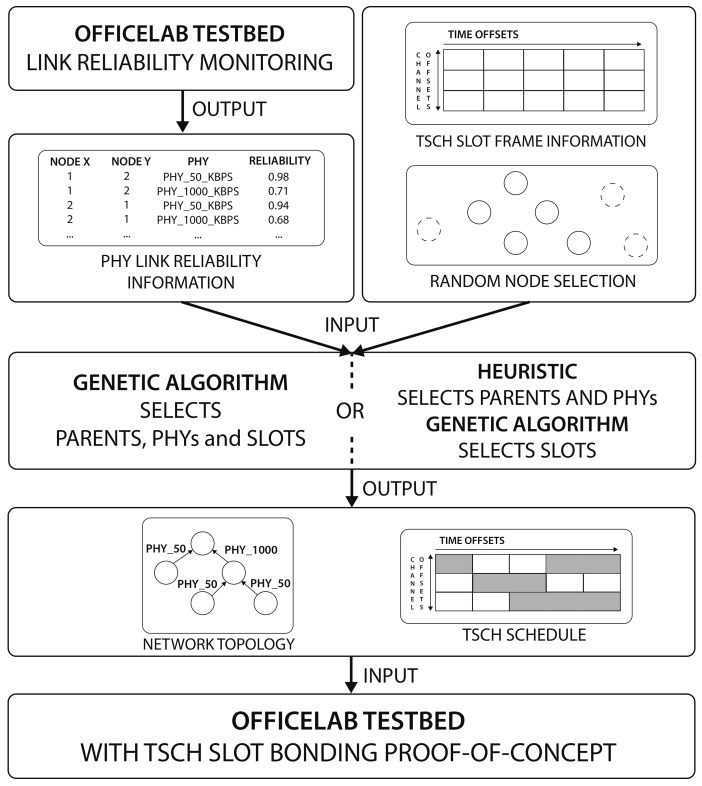
Flow diagram illustrating the testbed evaluation process. The testbed link monitoring information was used as input for the heuristic (with the GA for slot allocations) or the GA, and the outcomes of those schedulers were loaded on the sensor nodes in the testbed.

The testbed evaluation was a 3-step process as illustrated in Figure 7. First, using the slot bonding implementation, the link reliability between all nodes was monitored, in both directions, for the 50 kbps and 1000 kbps PHYs (as listed in Section 4.2). Every link was monitored for 300 s, transmitting a packet of 127 bytes every second, and its reliability was defined as the average $\frac{num\_ACKs}{num\_TX}$ of five consecutive observations, each over a time span of 60 s. The monitored link reliability values are publicly available (https://github.com/imec-idlab/officelab-reliabilities, accessed on 9 May 2021). Links between nodes that did not connect or became disassociated during the experiment were assigned a reliability value of 0. Two different scenarios were considered, each with 12 nodes as illustrated in Figure 5: scenario 1 included the 11 grey nodes and the blue node while scenario 2 included the 11 grey nodes and the green node. It turned out that, in normal conditions, the 1000 kbps PHY was able to connect (almost) all nodes with a very good reliability. Therefore, to better emulate an industrial environment in which the reliability of different network links can heavily vary and/or some nodes can not connect with certain PHYs, different transmission powers were used for the 2 PHYs and a part of the 1000 kbps PHY links was disabled in both scenarios. For the 50 kbps PHY, the transmission power was set to 14 dBm, while for the 1000 kbps PHY it was set to 0 dBm. Additionally, uniformly at random, 70% of the 1000 kbps PHYs links of each node (i.e., assigning links a reliability value of 0) were disabled. The goal of the monitoring step was to determine the link reliability values of all nodes to each other for each PHY, as these would be used by the heuristic to make the parent and PHY selections. While this monitoring step simplified our evaluation of the heuristic, in an integrated solution this could be replaced with run-time statistics as explained in Section 3.6. As such, afterwards, these reliability values were fed, together with the slotframe information and the selected set of nodes (including a randomly selected root node), to the GA scheduler. In the case of the heuristic, the GA only decided the slot allocations as the heuristic determined the parent and PHY selection for each node. Finally, the resulting network topologies with selected parents and PHYs and the TSCH schedules were configured on all selected nodes in the hardware testbed and the results were monitored for 300 ms.

**Figure 8:**
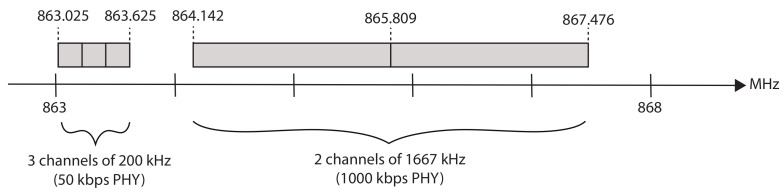
The channel allocation for the 50 kbps and 1000 kbps PHYs in the 863–868 MHz band, as used in the evaluation. The former uses 3 channels of 200 kHz while the latter uses 2 channels of 1667 kHz.

The slot bonding implementation deployed on the hardware testbed had a channel allocation of three channels of 200 kHz and two channels of 1667 kHz for the 50 kbps and 1000 kbps PHY, respectively, without frequency overlap in the 863–868 MHz band, as shown in Figure 8. The number of channels was limited to speed up the network bootstrap process. Two different slotframe lengths of 261 ms (i.e., 29 slots of 9 ms) and 423 ms (i.e., 47 slots of 9 ms) were considered. The nodes can actually only use 17 and 36 slots respectively in each slotframe: eight slots were included to facilitate two shared bonded slots (of four regular slots each) that use the 50 kbps PHY and padded the resulting length with unused extra slots to become a prime number of slots (to achieve pseudo-random channel hopping). The shared slots were placed at the beginning of each slotframe and were used to broadcast the TSCH Enhanced Beacon (EB) messages that advertised the network (every 7 s). The 6top Protocol (6P) layer and the Contiki-NG *NullNet* option were disabled, meaning there was no network or any higher layer functionality. All network topologies and schedule allocations were statically configured at the MAC layer, using the parent and PHY selections and the TSCH schedule provided by the heuristic and/or GA. The queue size was set to eight packets. The maximum number of allowed transmission opportunities per packet was set to four, as advised by the 6TiSCH Minimal Configuration [18]. Every node transmitted a packet of 127 bytes every slotframe.

For the configuration of the GA (i.e., operators, number of generation and mutation and cross-over probabilities), the reader is referred to our previous work as the GA configuration is the same [7]. However, the GA (and the proposed heuristic) now use the 50 kbps and 1000 kbps PHYs. Subsequently, the feasibility algorithm that determines the TSCH schedule was extended to be able to allocate bonded slots in the non-overlapping channels reserved for the respective PHYs. Additionally, the algorithm completely avoids interference between nodes by not allowing simultaneous transmissions using the same frequency channel.

The simulator results were averaged over 20 iterations, while the testbed results were averaged over 12 iterations. The used box plot graphs show the the first quartile value (i.e., Q1, the bottom of the box), the third quartile value (i.e., Q3, the top of the box), the minimum value (i.e., the lower whisker of the box plot, which equals Q1−1.5·IQR with IQR being Q3−Q1), the median value, the maximum value (i.e., the top whisker, which equals Q1+1.5·IQR) and the mean value (i.e., denoted by x). Outliers are defined as values smaller or larger than the minimum or maximum values. If there are outliers, these are denoted by the diamond shapes.

### 5.2. Simulator Results

**Figure 9:**
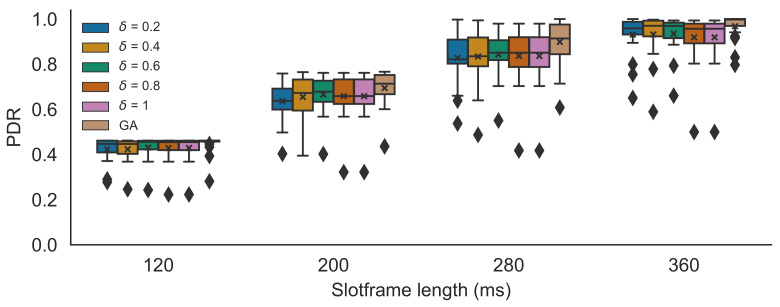
PDR simulation results for different δ values for different slotframe lengths, comparing the heuristic with the GA scheduler. The x denotes the mean value. The diamond shapes represent outliers that are values smaller than the minimum (i.e., the lower whisker) or larger than the maximum value (i.e., the top whisker).

**Figure 10:**
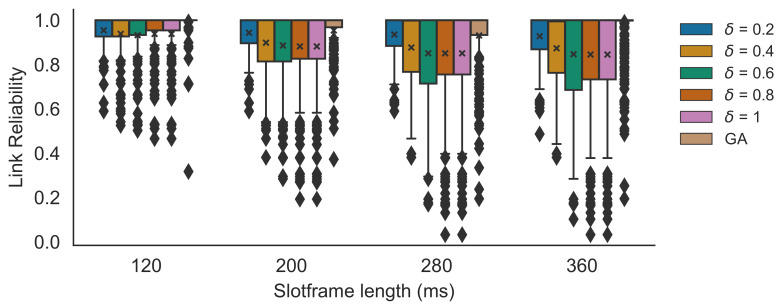
Mean link reliability values for different slotframe lengths, for different δ values of the heuristic. The x denotes the mean value. The diamond shapes represent outliers that are values smaller than the minimum (i.e., the lower whisker) or larger than the maximum value (i.e., the top whisker).

First, the heuristic for different δ values was compared to the GA scheduler in the TSCH simulation experiments with 14 nodes. The results are shown in Figure 9. The PDR results were compared for different slotframe lengths of 120 ms, 200 ms, 280 ms and 360 ms, and for δ values ranging from 0.2 to 1.0. When δ=1.0, the heuristic will pick for each possible parent the fastest available PHY. It is clear that the performance of the heuristic approximates the performance of the GA for all slotframe lengths. The PDRs of the GA scheduler were 0.446 (120 ms), 0.694 (200 ms), 0.898 (280 ms) and 0.968 (360 ms). While the performance of all δ values was very similar for all slotframe lengths, when δ=0.6 the PDRs were the highest with 0.431 (i.e., a decrease of 3.4% of the GA’s performance), 0.666 (i.e., a 4% decrease), 0.843 (i.e., a 6.1% decrease) and 0.935 (i.e., a 3.4% decrease), respectively. The average deviation in PDR values, observed between the heuristic and its near-optimal counterpart, was 0.052 and the maximum of all deviations was 0.331, when including all slotframe sizes and all δ values. When only taking into account δ=0.6, the average observed PDR difference was 0.044 and the maximum was 0.264. Figure 10 shows the reliability values for all links for the different δ values (with the mean reliability value denoted by an x). As expected (see Algorithm 1), the results show that with increasing δ values, the average link reliability value decreases. This decreasing reliability has an effect on the number of propagation failures, the number of needed retransmissions and, consequently, on the number of dropped packets (because of too many retransmission attempts). However, faster PHYs also need less transmission time and thus shorter bonded slots (i.e., less regular slots per bonded slot), leaving more free space in the schedule for extra retransmission slots or for other nodes to make slot allocations. As such, in this case, the best trade-off between using shorter/longer bonded slots and lower/higher reliability values was found when δ=0.6. This was confirmed by δ=0.6 consistently achieving the lowest total number of packet drops, including the dropped packets because of exceeding the retransmission threshold, but also the packets that were dropped immediately at the transmitter because there were not enough allocated slots to empty the queue on time.

### 5.3. Testbed Results

Here, the performance of the heuristic was evaluated on the testbed. First, an appropriate δ value was selected for the heuristic by comparing theoretical PDR values using the monitored reliability data. Afterwards, the PHY allocations for the different slotframe lengths were observed. Finally, the performance of the heuristic (with the selected δ) was compared to that of the near-optimal GA scheduler using our proposed slot bonding implementation on the testbed.

#### 5.3.1. δ Selection

**Figure 11:**
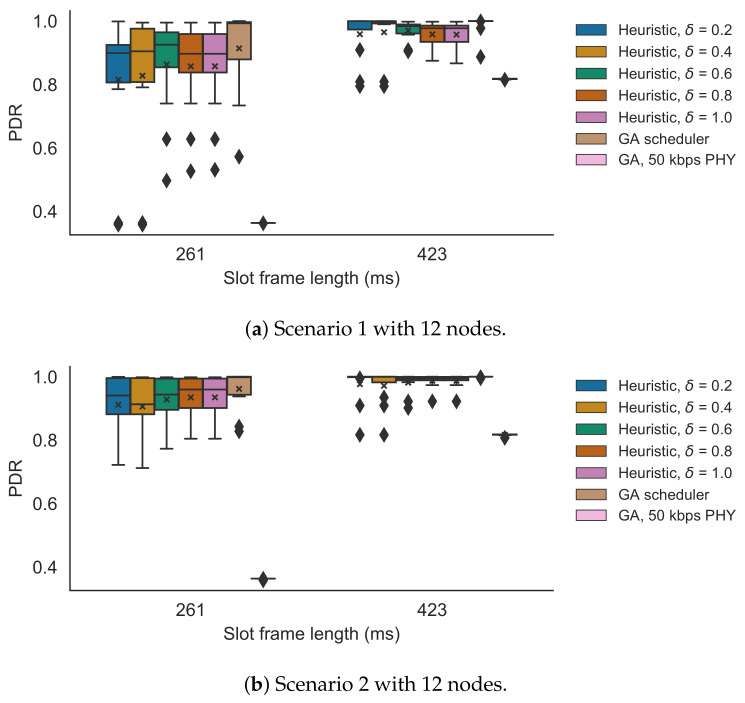
PDR values for different δ values for 261 ms and 423 ms slotframe lengths, comparing the heuristic with the GA scheduler and the GA scheduler that can only use the 50 kbps PHY. The x denotes the mean value. The diamond shapes represent outliers that are values smaller than the minimum (i.e., the lower whisker), or larger than the maximum, value (i.e., the top whisker).

**Figure 12:**
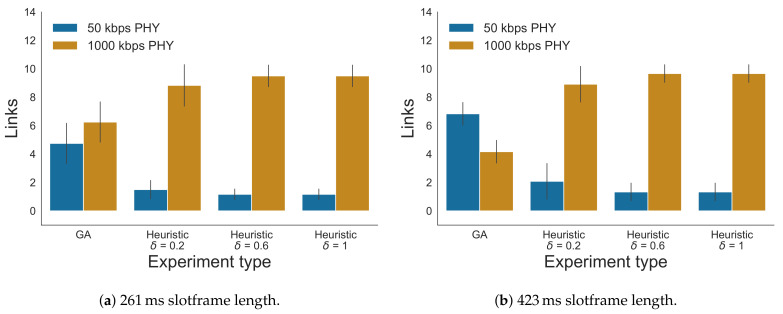
Average number of PHY allocations per iteration for the GA scheduler and the heuristic with different δ values, for scenario 2. The error bars in the figures represent the standard deviation.

First, the optimal δ values for the heuristic were determined, using the monitored link reliability values of the testbed. The expected number of delivered packets calculation was used, as described in the slot bonding problem formulation, to calculate the expected PDR values [7]. Figure 11 shows the comparison between different δ values, for the two network scenarios as discussed in Section 5.1.2, each for the two different slotframe lengths, 261 ms and 423 ms. It is clear from the results that the 261 ms slotframe was saturated, with all average PDR values being below 1. For scenario 1, with both the 261 ms and the 423 ms slotframes, the best performing δ was 0.6 with the PDRs being 0.86 and 0.97, while the PDRs of the GA were 0.91 and 0.99. Similar to the simulator results, the heuristic found the best trade-off between using fast PHYs with shorter bonded slots and lower reliability values when δ=0.6. When δ>0.6, the lower reliability actually started deteriorating the overall PDR. The average observed deviation in PDR values between the heuristic and its near-optimal counterpart was 0.05 and the maximum of all deviations was 0.37 in scenario 1, when including both slotframe sizes and all δ values. When only taking into account δ=0.6, the average observed difference was 0.04 and the maximum was 0.11. For scenario 2, the heuristic actually performs best when δ=0.8 with the average PDRs being 0.93 and 0.98 compared to the 0.96 and 1.0 PDRs of the GA. So again, the performance of the heuristic is very close to that of the centralized GA scheduler. For this scenario, the average deviation in PDR values between the heuristic and its near-optimal counterpart was 0.03 and the maximum of all deviations was 0.18, when including both slotframe sizes and all δ values. When only looking at δ=0.8, the average observed difference was 0.02 and the maximum was 0.09. The results show that the optimal δ was different for scenario 1 (i.e., δ=0.6) and scenario 2 (i.e., δ=0.8) (although the differences between the PDR results were small for both δ values in both scenarios). This is explained by the fact that the enabled 1000 kbps PHY links in scenario 1 showed lower link reliability values, when compared to the values of the enabled links in scenario 2 (as explained in Section 5.1.2, a random subset of the 1000 kbps links was disabled in each scenario). Therefore, in scenario 1, a δ>0.6 affects the PDR results more severely than in scenario 2 as it introduces less reliable links in the topology. Figure 12 shows the average number of times per iteration each PHY was used for a link for the GA scheduler and the heuristic with increasing δ values for scenario 2 for both slotframe lengths. The PHY allocations of scenario 1 are not shown as these were similar to those of scenario 2. Only links of nodes for which a packet could reach the root (i.e., there is at least one allocated slot for every link along the path towards the root) are shown. While the heuristic clearly prefers the faster 1000 kbps PHY (i.e., if the link is reliable enough, as defined in Algorithm 1), the GA also seems to focus on allocating 50 kbps PHYs. In the case of the heuristic, when δ increases, the number of 1000 kbps PHYs also increases and the number of used 50 kbps PHYs decreases. When comparing both slotframes, it is clear that for the longer slotframe length of 423 ms, it is possible to allocate more 50 kbps PHYs because there is more free space, leading to higher PDR values, as observed in Figure 11.

The PHY allocation and theoretical PDR results show that the heuristic generally performs well in terms of PDR but that the optimal δ value is dependent on the specific environment. Simultaneously, the results also clearly indicate that the heuristic does not necessarily need the most reliable links to perform well, but can benefit from using faster and less reliable PHYs that leave more free space for possible extra slots for retransmissions or other nodes’ allocations.

Additionally, Figure 11 shows the benefits of using multiple PHYs when only allowing the 50 kbps PHY results in PDRs of 0.36 and 0.82 for scenario 1 and PDRs of 0.36 and 0.82 for scenario 2, for the 261 ms and 423 ms slotframe, respectively. This is explained by the fact that the 50 kbps PHY requires bonded slots with a length of four regular slots, thereby occupying a lot of space in the schedule per transmission attempt and not allowing sufficient free space for extra slots or other nodes to allocate slots. The results for only using the 1000 PHY are not shown as that did not allow for fully connected topologies.

#### 5.3.2. Testbed Validation

**Figure 13:**
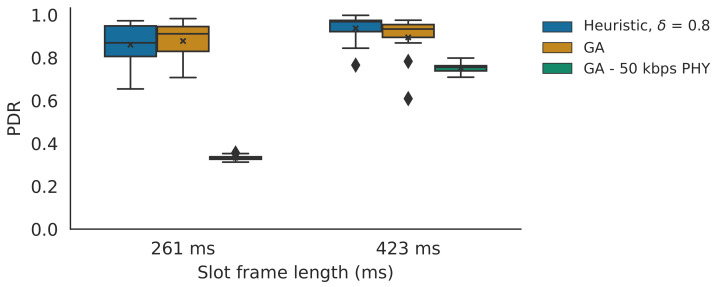
The PDRs values for the testbed experiments, showing the results for the heuristic, the GA and the GA that can only use the 50 kbps PHY. The x denotes the mean value. The diamond shapes represent outliers that are values smaller than the minimum (i.e., the lower whisker), or larger than the maximum, value (i.e., the top whisker).

Testbed experiments were carried out using the nodes of scenario 2 on the hardware testbed, using the proposed slot bonding implementation. Figure 13 shows the results for the heuristic (with δ=0.8), the multi-PHY GA and the single-PHY GA that can only use the 50 kbps PHY.

In general, it was observed that the testbed results approximate the theoretical values shown in Figure 11b. As expected, with the longer slotframe length, the mean PDR values also increased compared to the shorter slotframe length. More specifically, in the case of the 261 ms slotframe length, the GA performed better than the heuristic with PDRs of 0.88 and 0.86, respectively, meaning there was only a 2.3% decrease in performance. The GA that can only use the 50 kbps PHY was outperformed by its multi-PHY counterparts as it only achieved a PDR of 0.33 (i.e., the GA and the heuristic increased on this performance with 166.7% and 160.6%, respectively). When using a 423 ms slotframe length, the heuristic actually performed slightly better than the GA with PDRs of 0.94 and 0.88, and both again outperformed the single-PHY GA that had a PDR of 0.75 (i.e., the GA and the heuristic increased on this performance with 25.3% and 17.3%, respectively). The heuristic performing better than the multi-PHY GA is explained by the observation of link reliability values during these multi-hop experiments, which differed from the reliability values observed during the monitoring phase (on which the parent, PHY and slot allocation was based). More specifically, the monitoring results showed better performance mainly for the 50 kbps PHY compared to the values observed during the multi-hop experiments. As the GA more often uses 50 kbps PHY than the heuristic does, the PDRs of the GA was more negatively affected as a result. As such, these results actually motivate the integration of the heuristic in a routing protocol’s objective function with real-time link reliability monitoring to dynamically adapt to varying link conditions, as discussed in Section 3.6. Overall, the results clearly show the efficiency of the proposed parent and PHY selection heuristic.

## 6. Conclusions

This work further explored the support of multiple PHY layers in a single IEEE 802.15.4e TSCH slot bonding network. More specifically, the parent and PHY selection was investigated in such a multi-PHY network. First, a computationally efficient PHY and parent selection heuristic was proposed that can be integrated into a distributed routing protocol. The performance of this heuristic was initially evaluated via simulation and theoretical results, by comparing it against a near-optimal, but computationally complex, centralized scheduler. The results confirmed that the heuristic is capable of approximating the PDR results of the centralized scheduler. Second, an easy-to-configure TSCH slot bonding implementation was proposed in the latest version of the Contiki-NG IIoT operating system. Subsequently, the slot bonding implementation was used to confirm the PDR performance of the proposed heuristic in a real sensor testbed. In this testbed experiment, the proposed heuristic was at most 2.3% worse than the GA in terms of PDR. Both approaches outperformed the single-PHY solution, increasing PDR results by more than 160%.

## 7. Future Work

While during the experiments of the evaluation it was observed that the number of iterations the heuristic needed to converge was consistently below five (i.e., *c* in Section 3.5) and had no impact on its execution time, necessary future work will be the calculation of the exact upper bound of this number of iterations, to fully understand the limits of the heuristic. Furthermore, future work will be focused on a fully distributed implementation of the heuristic in the 6TiSCH stack with the low-power routing protocol RPL. Additionally, to further improve the performance of the heuristic in a completely distributed implementation, the trade-off between the current state of the node (e.g., amount of traffic, battery state and free schedule space) and the different characteristics of each possible PHY (e.g., achievable throughput, energy consumption and resource duration) should be carefully considered. Furthermore, while combining different PHYs with different transmission lengths, future implementation should also closely monitor and manage the frequency band duty cycle regulations, which is a requirement that is often overlooked.

## Figures and Tables

**Table 1:** The used PHYs configurations, together with their configured time slot length and number of bonded regular slots.

Modulation	Bandwidth (kHz)	Data Rate (kbps)	Time Slot Length (µs)	Bonded nr. of Slots
2-GFSK	200	50	36,000	4
4-GFSK	1667	1000	9000	1

**Table 2:** Possible channel allocations for 200 kHz and 1000 kHz channels in the European sub-GHz frequency band, as listed by Van Leemput et al. [4].

Start Freq.	End Freq.	Bandwidth	200 kHz	1667 kHz
863	868	5000	25	3
868	868.6	600	3	0
868.7	869.2	500	2	0
869.4	869.65	250	1	0
869.7	870	300	1	0
874	874.4	400	2	0
917.4	919.4	2000	10	1
			44	4

## Data Availability

The reliability values monitored at the imec Wireless OfficeLab (Ghent, Belgium), used in the evaluation section of this article, are publicly available at https://github.com/imec-idlab/officelab-reliabilities, accessed on 9 May 2021.
